# No effects on heart rate variability in depression after treatment with dorsomedial prefrontal intermittent theta burst stimulation

**DOI:** 10.48101/ujms.v128.8949

**Published:** 2023-03-27

**Authors:** Johan Bengtsson, Erik Olsson, Jonas Persson, Robert Bodén

**Affiliations:** a Department of Medical Sciences, Uppsala University, Akademiska Sjukhuset, Uppsala, Sweden; b Department of Women’s and Children’s Health, Uppsala University, Akademiska Sjukhuset, Uppsala, Sweden

**Keywords:** Depressive disorder, non-invasive brain stimulation, autonomic nervous system, sympathetic, parasympathetic, heart–brain connection

## Abstract

**Background:**

The purpose of this study was to investigate whether treatment of a depressive episode with intermittent theta burst stimulation (iTBS) over the dorsomedial prefrontal cortex (DMPFC) had any effects on heart rate variability (HRV). We also investigated if changes in HRV covaried with symptom change after iTBS and if HRV could predict symptom change.

**Methods:**

We included 49 patients with a current depressive episode. All were randomized to receive a double-blind treatment course with active or sham iTBS over the DMPFC. HRV data were obtained from 1 h of night data before and after the iTBS. The standard deviation of the RR interval (SDNN) was chosen as primary outcome measure. Depressive, negative, and anxiety symptoms as well as self-rated health were assessed by clinicians or by self-report.

**Results:**

The group×time linear mixed model revealed no effect of iTBS on SDNN (estimate = −1.8, 95% confidence interval [CI]: −19.9 to 16.2). There were neither correlations between HRV and depressive, negative, or anxiety symptom change after iTBS nor with self-assessed health. No predictive value of HRV was found.

**Conclusions:**

Treatment for depression with dorsomedial iTBS had neither negative nor positive effects on the cardiac autonomic nervous system.

## Introduction

Lower levels of heart rate variability (HRV) are generally associated with a wide range of diminished health aspects (1, 2). The interest for HRV in depression stems to a large extent from the association between depression and the risk of cardiovascular disease (3, 4). Unmedicated patients with depression exhibit significantly lower levels of HRV when compared to healthy controls, with effect sizes ranging from –0.096 to –0.462 (5). Medicated patients with depression have even lower HRV, probably partly due to the medications’ anticholinergic properties (6–10). It seems that hereditary factors do not contribute to the low HRV in depression, but psychosocial or life-style factors might explain part of the differences (5, 11, 12). Whether lower HRV mainly constitutes a state or a trait marker in depression is thus still an open question, and another way of approaching this issue has been to investigate if changes in depressive symptoms are accompanied by changes in HRV. For pharmacological or psychotherapeutic interventions in depression, it appears that symptom change is unrelated to changes in HRV, even if there are inconsistencies in the literature (8, 13). These findings favor the role of HRV as a trait marker in depression. Initiation or cessation of antidepressant pharmacological treatment has, however, been shown to decrease or increase HRV, respectively – further pointing out the important role of medication effects (10). Repetitive transcranial magnetic stimulation (rTMS) (14, 15) is a non-pharmacological treatment option for depression and might therefore provide an opportunity to study depressive symptom change and HRV without confounding medication effects. Various rTMS treatment protocols are being used, of which intermittent theta burst stimulation (iTBS) (16) has been of special interest since it can reach the same efficacy with a shorter duration of the daily treatment protocol (17). The most commonly used treatment target for rTMS in depression is the dorsolateral prefrontal cortex (DLPFC), which is part of the central autonomic modulation network, also including the dorsomedial prefrontal cortex (DMPFC), insula, amygdala, and other structures (18). The anterior cingulate cortex (ACC) is another area implicated in central autonomic modulation, and it is located just beneath the DMPFC (19). Using angled magnetic stimulation coils allows for modulating the ACC when applied over the DMPFC (20). It could therefore be expected that stimulation of both the DLPFC and the DMPFC would affect cardiac autonomic regulation. Indeed, while rTMS seems to increase HRV during the actual stimulation in healthy individuals (21–24), there is a scarcity of studies on the cardiac autonomic effects of rTMS in depression following a full treatment course. An open study reported increased HRV after rTMS (25) but the only sham-controlled study measuring the concomitant effect of iTBS found no enduring effects on HRV, albeit acute changes during the iTBS session, thus converging with the findings in healthy individuals (26). Broadening the diagnostic perspective to schizophrenia, a recent large, multi-center and sham-controlled study found no effects of an rTMS treatment course on heart rate (27). Regarding HRV and concomitant symptom change in depression after rTMS, there are only few reports of such associations. The open study mentioned above reported a correlation between post-treatment depression scores and post-treatment HRV, but there were no comparisons with baseline values (25). Another study reported a non-significant correlation between heart rate decelerations during the first treatment session and greater symptom reduction (26). This finding has been argued to open up for heart rate decelerations as a predictor for treatment response in depression (28), but to the best of our knowledge, there are no studies on HRV as a predictor for treatment response after rTMS. The results of HRV as a predictor of treatment response for other treatment options in depression have hitherto been mixed (29–31).

In summary, there is only one open study reporting an increase in HRV after a full treatment session of rTMS. This finding would be strengthened if replicated with a sham-controlled design. Also, since other treatment options for depression, such as tricyclic antidepressant agents (TCAs), clearly diminish HRV, a lack of such negative treatment side effects of rTMS would be beneficial. The present study aimed at investigating the effect on HRV of a treatment course with iTBS, targeting the DMPFC in patients with depression. Further aims were to investigate the correlation between symptom change and change in HRV and also to investigate whether baseline HRV would predict symptom change.

## Materials and methods

### Participants

**Figure 1:**
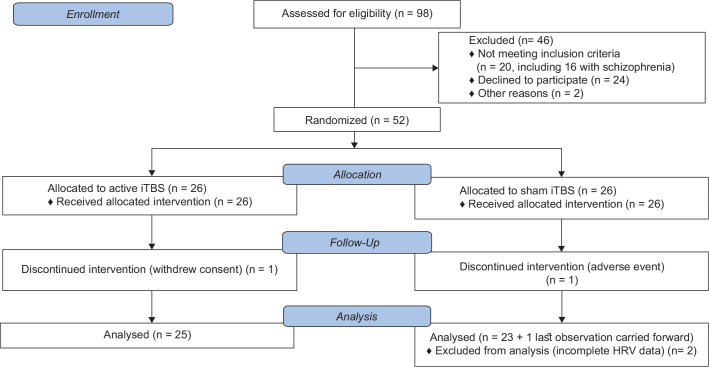
CONSORT flow chart of the subsample constituting the current study.

The 49 participants were a subsample of patients from a randomized controlled trial (clinicaltrials.gov identification number NCT02905604) of dorsomedial prefrontal iTBS for negative symptoms in schizophrenia or depression, and from an add-on brain-imaging study (32, 33). The transdiagnostic approach was based on the observed overlap between negative and depressive symptoms (34). Inclusion criteria were originally designed for the above-mentioned trial, but for the current study, they were as follows: written informed consent, being 18–59 years old, a diagnosis of uni- or bipolar depression established by a psychiatrist in clinical routine care and verified through a Mini International Neuropsychiatric Interview (M.I.N.I., Swedish translation of version 6.0.0) (35), less or equal than 40 points on The Motivation and Pleasure Scale-Self-Report (MAP-SR) (36), and no changes in medication during the past month. Exclusion criteria were standard rTMS such as metals implanted in the head, epilepsy, pacemakers, vagus nerve stimulators, medication pumps, etc. (37). Other exclusion criteria were any condition implicating a substantial risk of non-compliance or loss of follow-up, active substance use disorder (except nicotine and caffeine), and pregnancy. The patients with schizophrenia recruited to the main trial were excluded from the current study (*n* = 16). See Figure 1 for a flow chart. The study was conducted in accordance with the Helsinki declaration and approved by the Research Ethical Review Board in Uppsala. The trial was conducted between 2016 and 2020 at Uppsala university hospital in Sweden.

### rTMS procedures

The DMPFC was localized with neuronavigation (33) or defined as 25% of the distance between the nasion and the inion (*n* = 11). The iTBS was delivered with a magnetic stimulator Magpro X100. The coil was an angled combined active/placebo coil (Cool-DB80 A/P) with two identical sides, of which one side was shielded, so that the majority of the magnetic field did not reach the participant. Upon entering a randomization code into the stimulator, the operator was instructed by the software which side to position toward the participant’s head (tangentially to the scalp, handle toward the right side of the participant). Resting motor threshold was defined as the lowest magnetic stimulator output needed for a visually observable muscle contraction in the musculus extensor hallucis longus in the foot in 50% of the trials, determined by an automated maximum likelihood strategy (38, 39). The active iTBS was a modified version of earlier protocols (39, 40), with 20 trains of stimulation with right-left stimulation and 20 trains with left-right stimulation. A second identical treatment session was delivered after a 15 min break (41, 42). Stimulation was applied at 90% of resting motor threshold with a total of 2,400 pulses per day. The sham iTBS comprised an identical protocol but with the shielded side of the coil toward the participant, whereby only a weak magnetic field was applied. In addition, two transcutaneous electrical nerve stimulation (TENS) electrodes were placed under the coil of both the active and sham participants’ foreheads. In order to also mimic the sensation of the magnetic stimulation in the sham group, a current of maximum 4 mA (scaled to the magnetic stimulator output intensity) was delivered synchronous with the magnetic pulses. The treatment was delivered by a research nurse, and the symptom raters were not present during the treatment sessions. Stimulation intensity was ramped up to minimize discomfort, aiming at 10 consecutive weekdays of treatment at target intensity (32).

### Heart rate variability

The two-electrode heart rate recorder Firstbeat Bodyguard 2 (Firstbeat Technologies Ltd., Jyväskylä, Finland) was used to monitor heart rate. The device was attached across the chest by a research nurse on the morning the day before the first treatment day. After this visit, the participants wore the device until the next day, also during the night, and returned it when they came back for their first treatment session. The same procedure was repeated the day after the last treatment, 2 weeks after baseline. Sampling rate was 1,000 Hz with a resolution of 1 ms. Since the aim of this study was to assess the potential lasting effects on HRV, an hour of data were extracted during the night (midnight to 5 am). This hour was chosen from a period where heart rate seemed stable, when inspected visually. The chosen hour varied between subjects depending on visual inspection of where there were fewest artifacts. This hour could also vary between each participant’s baseline and follow-up data, but was aimed to be the same. Within the chosen time period, the following HRV metrics were calculated: the standard deviation of the RR interval (SDNN), the root mean square of successive RR interval differences (RMSSD), high frequency HRV (HF), low frequency HRV (LF), LF/HF ratio, and the RR triangular index (2). LF and HF were logaritmized. As an open study has previously reported changes in SDNN after rTMS, this metric was chosen as the primary outcome measure to enable comparisons (25). If artifact correction was necessary after visual inspection, the inbuilt algorithm in the software Kubios HRV Standard (version 3.0.2) was used. The algorithm compares the length of each interbeat interval (IBI) to an average of the surrounding IBIs and classifies IBIs that differ from a selected threshold as potential artifacts.

In a subset of patients (*n* = 37), we also extracted HRV data from a 5-min daytime period when the participants were resting, sitting in a supine position just after the device was attached. This data collection was performed both the day before the first treatment session (baseline) and 4 weeks after baseline. From this period, we extracted HF-HRV data. These data were used for a separate analysis of the lasting effects of iTBS on HRV. The data were only available in a subset of patients due to slightly different designs of the scheduled time frames of the main trial (32) and the add-on brain imaging study (33). This was done in order to evaluate the effect of iTBS on two different standard HRV metrics – SDNN and HF-HRV.

### Clinical assessments

Since the original trial aimed at treating negative symptoms with a transdiagnostic approach, patients were assessed at the baseline visit (the day before the first treatment day) by a trained physician with the Clinical Assessment Interview for Negative Symptoms (CAINS) (43, 44) and the Brief Psychiatric Rating Scale – extended (BPRS-E) (45). Participants also filled in self-reports: sociodemographic data, nicotine consumption, The Alcohol Use Disorders Identification Test (AUDIT) (46), The Drug Use Disorders Identification Test (DUDIT) (47), Montgomery-Åsberg Depression Rating Scale – Self-report (48), and self-assessed health with the EQ-VAS (49). The same interviews and symptom ratings were repeated the day after the last treatment session (i.e. 2 weeks from baseline), and also 4 weeks after baseline. After that, the concealed treatment allocation was unblinded, and participants who had been receiving sham iTBS were offered an open treatment course.

### Statistics

All data were assessed for normality by visual inspection of histograms, Q-Q plots, and with the Shapiro–Wilk test. Means and standard deviations were reported if variables were found to be normally distributed and medians and inter-quartile ranges if the data distributions were assessed to be skewed. To investigate differences in demographic variables between the groups, we used the independent-samples *t*-test for continuous normally distributed variables, Mann–Whitney U-test for continuous non-normally distributed variables, and chi square or Fisher’s exact test for dichotomous and rank variables.

In the primary analysis, we used a linear mixed model using maximum likelihood estimation with random intercept per subject to assess group and time effects of iTBS on SDNN (or HF-HRV in the subsample of 37 patients).

For the correlation between HRV metrics and symptom change, Pearson correlation coefficients were computed to assess correlations between delta values of symptoms and delta values of SDNN. The symptom ratings from the assessment 2 weeks after baseline were used, except for eight participants, where the ratings 4 weeks after baseline were used (due to slightly different time frames for the participants also participating in the brain imaging study mentioned above).

To assess the predictive value of baseline SDNN on symptom change, we used a linear regression model with delta values of symptoms as the dependent variable and baseline SDNN as the independent variable. Here, we only analyzed the active iTBS data from both the 25 participants receiving active iTBS at first and the data from the participants from the sham group who decided to enter an open phase with active iTBS (*n* = 17), resulting in a sample of 42 participants. The procedures for the iTBS sessions, HRV assessments, and symptom ratings were identical for both the active and sham groups. The symptom ratings from the assessment 2 weeks after baseline were used, except for six participants, where the ratings 4 weeks after baseline were used (due to the same reason as mentioned above). We assessed linearity between the variables through visual inspection. The presence of influential outliers was also inspected visually as well as with the Cook’s Distance. Multicollinearity was assessed with the variance inflation factor. The Durbin–Watson statistic was used to test the independency of the residuals’ values, and PP plots were used to inspect the distribution of the residuals. The residuals’ variance indicated no severe heteroscedasticity. SPSS version 26 was used for the statistical calculations.

## Results

### Demographics

All demographic data are presented in Table 1. Overall, the two randomized groups were similar, but there were more patients in the sham group with a significant difference regarding body mass index (BMI) and prescription of positive chronotropic drugs. However, there were no significant differences in baseline SDNN between the groups (independent *t*-test, *t* = 0.29, *P* = 0.774). For BMI, a linear regression model revealed no significant impact of BMI on baseline SDNN (*β* = 0.016, *P* = 0.914). There was one participant in the active group on antidiabetic treatment.

**Table 1:** Demographic and clinical characteristics (*n* [%] if not stated otherwise).

Variable	Active (*n* = 25)	Sham (*n* = 24)	*P* ^a^
Age (years), median (IQR)	27 (14)	27 (12)	0.674
Sex, *n* (female/male)	14/11	14/10	0.869
BMI (kg/m^2^), median (IQR)	25 (12)	23 (7)	0.040
Bipolar depression	1 (4)	3 (13)	0.349
Comorbidity anxiety disorders	9 (36)	11 (46)	0.484
Comorbidity neuropsychiatric disorders	6 (24)	8 (33)	0.470
Supported housing	2 (8)	5 (21)	0.247
**Level of education**			
Primary school	3 (12)	5 (21)	0.463
High school	12 (48)	15 (63)	0.308
Higher education	10 (40)	3 (13)	0.051
In current employment or studying	15 (60)	15 (63)	0.858
Sheehan Disability Scale, median (IQR)	19 (10)	18 (11)	0.865
Nicotine use^b^	7 (28)	9 (38)	0.478
AUDIT total score, median (IQR)	3 (3)	5 (6)	0.643
DUDIT total score, median (IQR)	0 (8)	0 (3)	0.940
PSQI sleeping time (h), mean (SD)^c^	8 (2)	7 (2)	0.096
Baseline HRV data starting time, mean time a.m. (SD in min)	2:28 (68)	2:39 (58)	0.529
Follow-up HRV data starting time, mean time a.m. (SD in min)	2:38 (71)	2:42 (67)	0.701
Difference between starting times (baseline – follow-up HRV data), min, mean (SD)	22 (29)	27 (41)	0.611
**Prescriptions**			
SSRI	6 (24)	5 (21)	0.791
SNRI	7 (28)	9 (38)	0.478
TCA	5 (20)	2 (8)	0.417
Antidepressant combination	10 (40)	7 (29)	0.426
Lithium	6 (24)	4 (17)	0.725
Antipsychotic	4 (16)	5 (21)	0.725
Positive chronotropic drug^d^	2 (8)	9 (38)	0.018
Negative chronotropic drug^d^	1 (4)	4 (17)	0.189

IQR: interquartile range; BMI: body mass index; SD: standard deviation; AUDIT: Alcohol Use Disorders Identification Test; DUDIT: Drug Use Disorders Identification Test; PSQI: Pittsburgh Sleep Quality Index; SSRI: selective serotonin reuptake inhibitor; SNRI: Serotonin and Norepinephrine Reuptake Inhibitors; TCA: tricyclic antidepressant agent; HRV: heart rate variability.

a Independent-samples t-test/Mann–Whitney U-test for continuous variables, chi square for dichotomic variables, and Fisher’s exact test if n < 5 in any cell with dichotomic variables.

b Tobacco or Swedish snuff.

c Two missing.

d Positive chronotropic drugs included dexamphetamine, levothyroxine, and methylphenidate. Negative chronotropic drugs included betablockers, guanfacine, and thiamazole.

### HRV and symptom data

See Table 2 for HRV and symptom burden data. The sham group had lower heart rate both at baseline and at follow-up. The difference was borderline significant at baseline (independent sample *t*-test, *P* = 0.052) and significant at follow-up (*P* = 0.028). There were no significant differences in SDNN between the groups, neither at baseline nor at follow-up.

**Table 2:** Effect of iTBS on HRV and symptoms.

Variable	Baseline		Follow-up		Group×time Estimate	CI lower	CI upper
	Active (*n* = 25)	Sham (*n* = 24)	Active (*n* = 25)	Sham (*n* = 24)			
IBI (ms)	868.6 (147.5)	941.0 (132.5)	857.6 (132.5)	933.3 (131.0)	−3.3	−110.3	103.6
HR (bpm)	71.0 (11.8)	65.0 (9.0)	71.4 (9.9)	65.4 (8.5)	0.0	−7.8	7.8
**SDNN (ms) (chosen outcome)**	37.7 (26.5)	39.9 (27.1)	35.6 (18.2)	39.6 (18.7)	−1.8	−19.9	16.2
RMSSD (ms)	37.1 (35.2)	40.8 (39.5)	32.9 (23.2)	37.6 (27.1)	−1.0	−26.0	24.1
logLF	6.1 (1.3)	6.2 (0.9)	6.1 (1.4)	6.4 (0.8)	−0.2	−1.1	0.7
logHF	5.7 (1.6)	5.8 (1.6)	5.7 (1.4)	5.8 (1.4)	0.0	−1.2	1.2
LF/HF	2.1 (1.9)	2.3 (1.7)	2.1 (2.1)	2.8 (2.2)	−0.5	−2.0	1.1
RR triangular index	10.2 (7.8)	10.9 (7.5)	9.7 (4.6)	10.5 (5.2)	−0.3	−5.3	4.8
Artifacts corrected (% of beats removed)	1.3 (5.7)	0.1 (0.2)	0.4 (1.5)	0.3 (0.4)	−1.1	−3.4	1.3
**Symptom ratings**							
CAINS total score	27.4 (7.8)	31.1 (7.4)	21.2 (9.8)	28.6 (8.2)	−3.7	−10.2	2.8
MADRS-S total score	29.2 (8.3)	30.1 (7.3)	24.8 (9.6)	28.0 (9.4)	−2.3	−9.1	4.6
BPRS anxiety subscalea	6.7 (2.9)	7.7 (2.6)	6.0 (2.6)	6.4 (2.6)	0.7	−1.5	2.8
EQ-VAS	33.8 (16.8)	34.8 (15.0)	43.8 (19.3)	40.4 (16.4)	4.4	−8.9	17.8

Means and standard deviations for descriptive values. Estimates and 95% confidence intervals for linear mixed model results.

iTBS: intermittent theta burst stimulation; HRV: heart rate variability; CI: confidence interval; IBI: Interbeat Interval; ms: milliseconds; HR: heart rate; bpm: beats per minute; SDNN: standard deviation of the RR interval; RMSSD: square root of the mean squared differences of successive RR intervals; RR: ; logLF: logarithmic low frequency; logHF: logarithmic high frequency; CAINS: Clinical Assessment Interview for Negative Symptoms; MADRS-S: Montgomery Asberg Depression Rating Scale self-rating; BPRS: Brief Psychiatric Rating Scale; EQ-VAS: EuroQoL Group Visual Analogue Scale.

^a^Sum of items Anxiety and Tension.

Regarding the effect of iTBS on SDNN, there was no interaction effect of group×time (estimate = −1.8, 95% confidence interval [CI]: −19.9 to 16.2). No effects on the secondary HRV variables were noted.

**Figure 2:**
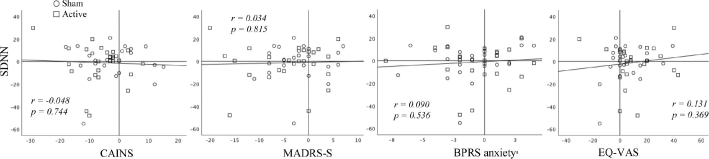
Scatter plots for delta values of symptoms and delta values of HRV. Note that a positive value of EQ-VAS indicates health improvement, whereas positive values on the other scales represent increased symptom load. *r = Pearson correlation coefficient for the regression line (similar results using Spearman’s correlation coefficients)*. *HRV:* heart rate variability; SDNN: standard deviation of the RR interval; CAINS: Clinical Assessment Interview for Negative Symptoms; MADRS-S: Montgomery Asberg Depression Rating Scale self-rating; BPRS: Brief Psychiatric Rating Scale; EQ-VAS: EuroQoL Group Visual Analogue Scale. ^a^Sum of items Anxiety and Tension.

There were no significant symptom reduction and no correlations between change in SDNN and symptom change on CAINS, MADRS-S, BPRS anxiety subscale, or self-assessed health. In the correlation analyses, we analyzed the active and sham groups together, but subgroup analyses of the active and sham group separately revealed no differences (data not shown). See Figure 2 for a graphical presentation of the correlations.

Regarding the predictive value of SDNN, there was no effect of baseline values of SDNN on any of the symptom categories, see Table 3.

**Table 3:** HRV as a predictor for symptom change after iTBS.

Variable	SDNN		
	*B*	*beta*	*P*
CAINS	0.061	0.146	0.355
MADRS-S	−0.022	−0.055	0.732
BPRS anxiety^a^	0.004	0.031	0.843
EQ-VAS	0.063	0.078	0.621

Linear regression results. Dependent variable: delta values of symptoms (*n* = 42).

HRV: heart rate variability; iTBS: intermittent theta burst stimulation; SDNN: standard deviation of the RR interval; CAINS: Clinical Assessment Interview for Negative Symptoms; MADRS-S: Montgomery Asberg Depression Rating Scale self-rating; BPRS: Brief Psychiatric Rating Scale; EQ-VAS: EuroQoL Group Visual Analogue Scale.

a Sum of items Anxiety and Tension.

The main analysis of the effect of iTBS on HRV was also run in the subsample of patients (*n* = 37) with 5 min daytime resting HRV data available (before the treatment course and 4 weeks after baseline). In this model, we used logarithmic HF-HRV as the dependent variable. The results did not differ substantially from the main analysis (estimate = −0.24, 95% CI: −1.60 to 1.12).

## Discussion

To the best of our knowledge, this is so far the largest study investigating the effect on HRV after a treatment course of iTBS targeting the DMPFC in depression. We did not detect any significant effects on the HRV metrics. Neither there were any correlations between change in HRV and symptom change detected, nor there was any predictive value of baseline HRV on symptom change.

First, it has to be pointed out that we specifically assessed HRV before and after the iTBS treatment course and therefore cannot draw any conclusions about whether iTBS has an acute and transient effect on HRV. Comparisons with studies on healthy individuals are hindered due to the fact that no healthy individuals receive longer treatment courses. Of the sham-controlled studies on healthy individuals, there have been no effects that lasted over time (22, 50–54). Regarding depression, the only earlier sham-controlled study investigated the acute effects of iTBS in 15 patients with depression and mainly focused on heart rate decelerations, which complicates comparisons with our investigation of the enduring effects of a treatment series with iTBS (26). Concerning HRV in that study, it was found that HRV was significantly more affected by active iTBS than sham. The sham condition though (shielded coil placed over the vertex) did not produce any comparable pain stimuli as the active iTBS, which leaves the possibility to open that these observations are connected to the pain effects of the stimuli, since arousal effects have been shown to be important (55). The treatment target in the same study was the DLPFC and not the DMPFC as in our study. Even if both of these structures are connected to the ACC, which is an important component of the central autonomic network (18, 19), it cannot be ruled out that differential locations implicate separate effects on HRV. Indeed, a study from the same research group indicates this (56). Nevertheless, taken together, these differences hamper further comparisons between the two studies.

As for non-sham-controlled studies of the effect of rTMS on HRV, an open study found increased SDNN between baseline and follow-up 2 weeks later in 30 patients with depression treated with rTMS (targeting DLPFC using 5-cm-rule, 15 Hz, 100% of resting motor threshold (rMT), 1,500 pulses per session). They assessed HRV before and after the treatment, which possibly eliminates the potential confounding effects of the simultaneous pain or sensational stimuli of rTMS. However, since the study was not sham-controlled, there can be no speculations of the specific effects of rTMS on HRV (25). In our study, we did not observe any changes at all, regardless of active or sham treatment. However, our studies differ in many aspects. The sample in the study by Udupa et al. consisted of drug-naïve patients without psychiatric or somatic comorbidities and with a milder depression score rating at baseline, whereas our sample consisted of patients on medication, with comorbidities and higher baseline depression rating scores. The HRV assessments also differ in that we used an hour of night data, whereas their study used 5 min of resting data as well as different assessment conditions. Comparisons between the studies must therefore be cautious.

Regarding the correlation between HRV change and symptom change, we did not find any such correlations. In the literature, this question has been of interest as a way of approaching the causal interactions between HRV and symptom change. In an open study of 27 patients receiving rTMS, there was no correlation between symptom reduction and HRV or other autonomic parameters (57). Adding three patients to the same sample yielded a correlation between follow-up values of LF/HF and depressive symptoms at follow-up, but there were no comparisons with baseline values (25).

We could not find any predictive value of HRV regarding treatment response. Here, we analyzed all open treatment sessions in our study (*n* = 42). Earlier efforts of using HRV as a predictor of treatment response in depression have not led to any conclusive results, even if there are positive findings of other treatment modalities scattered in the literature (29, 30). Results from the only sham-controlled rTMS study investigating this issue in 15 patients with depression could not find any correlations between decelerations of heart rate early in the treatment course and clinical response after the treatment, even if there was a trend toward such an effect (26). More research is needed before HRV can achieve acceptable prediction properties in the field of depression treatment.

There are some limitations of our study. Even though our sample is, to our knowledge, the so far largest sample investigating the effects of a sham controlled treatment course of iTBS on HRV, it is still a small sample size. It has been argued that interpreting CIs is better than post-hoc analyses to judge the robustness of ‘null’ results (58). Our results yielded quite wide CIs for all the outcome measures (see Table 2). The CIs for SDNN spanned from a decrease by approximately 20 ms to an increase by 16 ms. It can therefore be stated that iTBS at least does not affect SDNN more or less than that.

Regarding the HRV data extraction, we could not ascertain that the participants actually slept. However, the time frame of midnight to 5 am is a time when most people do sleep, and the participants in both groups reported a sleeping time of around 7 h. Furthermore, the mean starting time for the HRV data did not differ between the groups. The baseline and the follow-up HRV data were in some cases extracted from different times during the night, but the mean difference was only 25 min in the whole sample (see Table 1). However, HRV might also fluctuate according to sleep phase, which we were unable to assess (59). This might have introduced uncontrolled variability and thus be a source for our negative findings. Our converging results from the subsample analysis of a standard 5 min HRV recording however strengthen our results somewhat. Future studies assessing HRV during night would merit from a more detailed sleep phase assessment.

We chose SDNN as our primary outcome. Our main interest has not been the parasympathetic nervous system in isolation, and SDNN originates from both sympathetic and parasympathetic signaling (60). SDNN is encouraged for use in longer recordings, even if a 24-h recording would have been preferable. Furthermore, it has the advantage of being more easily interpreted and is also used in the important prediction study of HRV and cardiovascular risk (61). In the main analysis, we also analyzed a subsample (*n* = 37) using the vagal measure of HF-HRV, and the results did not differ to that of SDNN.

Some argue that baseline statistical comparisons between the groups in randomized data are not necessary (62). However, if the study sample is restricted, there is still a risk of imbalance between the groups. In the current study, there were no significant differences between the treatment groups regarding known baseline factors that might affect the outcome. We therefore refrained from controlling for factors such as age and sex.

The participants’ medications were quite diverse, possibly reflecting the heterogeneity of the treatment-resistant depression concept and the aim of recruiting a clinically representative sample. Importantly, all drug regimens were kept stable during this study, and even though these medications could have an impact on HRV, it is therefore unlikely that they would contribute to a change in HRV during the study period. Some drugs prescribed (most notably TCAs) could cause a high occupancy of the muscarinic receptors in the heart and might thereby diminish the vagal possibilities to modulate heart rate, thus rendering these participants unable to change their HRV. There were however no substantial differences in TCA prescription between the active and the sham groups.

Eight participants’ symptom ratings originated from 4 weeks after baseline rather than 2 weeks after baseline. However, at least some of the symptoms assessed are not subject to rapid fluctuations but rather quite stable. Furthermore, excluding these participants did not materially change the results (data not shown).

The use of delta values in correlational analyses may introduce measurement error from both baseline and follow-up assessments with subsequent unreliability. Our results from these analyses are therefore to be interpreted with caution, and the reader is referred to Figure 1 for a visual interpretation.

Our sample consisted of patients recruited to a transdiagnostic study of the effects of iTBS on negative symptoms. The results are therefore not necessarily generalizable to a wider group of patients with depression. The patients who come in contact with rTMS are however often labeled as ‘treatment resistant’, and even if these patients can be defined in many ways (63, 64), our sample is probably comparable to this group. Some of these patients are already exposed to treatments with well documented effects on their cardiac autonomic nervous system functioning, for example, TCAs (6). Our results indicate that rTMS lacks such effects.

We conclude that treatment with dorsomedial iTBS did not increase HRV, but it did not have any negative effects on the cardiac autonomic nervous system.
